# *Rhizobiales*-Specific RirA Represses a Naturally “Synthetic” Foreign Siderophore Gene Cluster To Maintain *Sinorhizobium*-Legume Mutualism

**DOI:** 10.1128/mbio.02900-21

**Published:** 2022-02-08

**Authors:** Ke-Han Liu, Biliang Zhang, Bo-Sen Yang, Wen-Tao Shi, Yu-Fei Li, Yin Wang, Pan Zhang, Jian Jiao, Chang-Fu Tian

**Affiliations:** a State Key Laboratory of Agrobiotechnology, and College of Biological Sciences, China Agricultural University, Beijing, China; b MOA Key Laboratory of Soil Microbiology, and Rhizobium Research Center, China Agricultural University, Beijing, China; University of California, Los Angeles; University of Hawaii at Manoa

**Keywords:** horizontal gene transfer, petrobactin, soybean, symbiosis

## Abstract

Iron homeostasis is strictly regulated in cellular organisms. The *Rhizobiales* order enriched with symbiotic and pathogenic bacteria has evolved a lineage-specific regulator, RirA, responding to iron fluctuations. However, the regulatory role of RirA in bacterium-host interactions remains largely unknown. Here, we report that RirA is essential for mutualistic interactions of Sinorhizobium fredii with its legume hosts by repressing a gene cluster directing biosynthesis and transport of petrobactin siderophore. Genes encoding an inner membrane ABC transporter (*fat*) and the biosynthetic machinery (*asb*) of petrobactin siderophore are sporadically distributed in Gram-positive and Gram-negative bacteria. An outer membrane siderophore receptor gene (*fprA*) was naturally assembled with *asb* and *fat*, forming a long polycistron in S. fredii. An indigenous regulation cascade harboring an inner membrane protease (RseP), a sigma factor (FecI), and its anti-sigma protein (FecR) were involved in direct activation of the *fprA-asb-fat* polycistron. Operons harboring *fecI* and *fprA-asb-fat*, and those encoding the indigenous TonB-ExbB-ExbD complex delivering energy to the outer membrane transport activity, were directly repressed by RirA under iron-replete conditions. The *rirA* deletion led to upregulation of these operons and iron overload in nodules, impaired intracellular persistence, and symbiotic nitrogen fixation of rhizobia. Mutualistic defects of the *rirA* mutant can be rescued by blocking activities of this naturally “synthetic” circuit for siderophore biosynthesis and transport. These findings not only are significant for understanding iron homeostasis of mutualistic interactions but also provide insights into assembly and integration of foreign machineries for biosynthesis and transport of siderophores, horizontal transfer of which is selected in microbiota.

## INTRODUCTION

Iron is a common good for co-occurring community members in various niches (1). Abundant iron exists in the biosphere but mainly in the oxidized ferric (Fe^3+^) form that is insoluble under neutral and basic pH conditions (1). To utilize the available ferric iron, many bacteria secrete siderophores to scavenge Fe^3+^ by forming soluble ferric siderophore complexes, which can be then actively taken up via specific outer membrane receptors (Gram-negative bacteria) and various ABC transporters (both Gram-negative and Gram-positive bacteria) (2, 3). Hundreds of known siderophores fall under four main chemical classes, catecholate, hydroxamate, carboxylate, and phenolate, which are distinguished from each other on the basis of moieties chelating ferric iron (2). There are plenty of examples of uptake of ferric siderophore complexes as a public good by siderophore nonproducers and co-occurring cheating resistance mechanisms in siderophore producers such as secreting different siderophores (4–7). Notable variations in iron affinity of siderophores and condition-dependent stability of ferric siderophore complexes (3, 8) make siderophores a competitive trait of bacteria (9). This, in turn, drives horizontal transfer of various siderophore biosynthesis and/or transport genes among bacteria (10); however, the regulatory integration process during or post-horizontal transfer is poorly understood.

**FIG 1:**
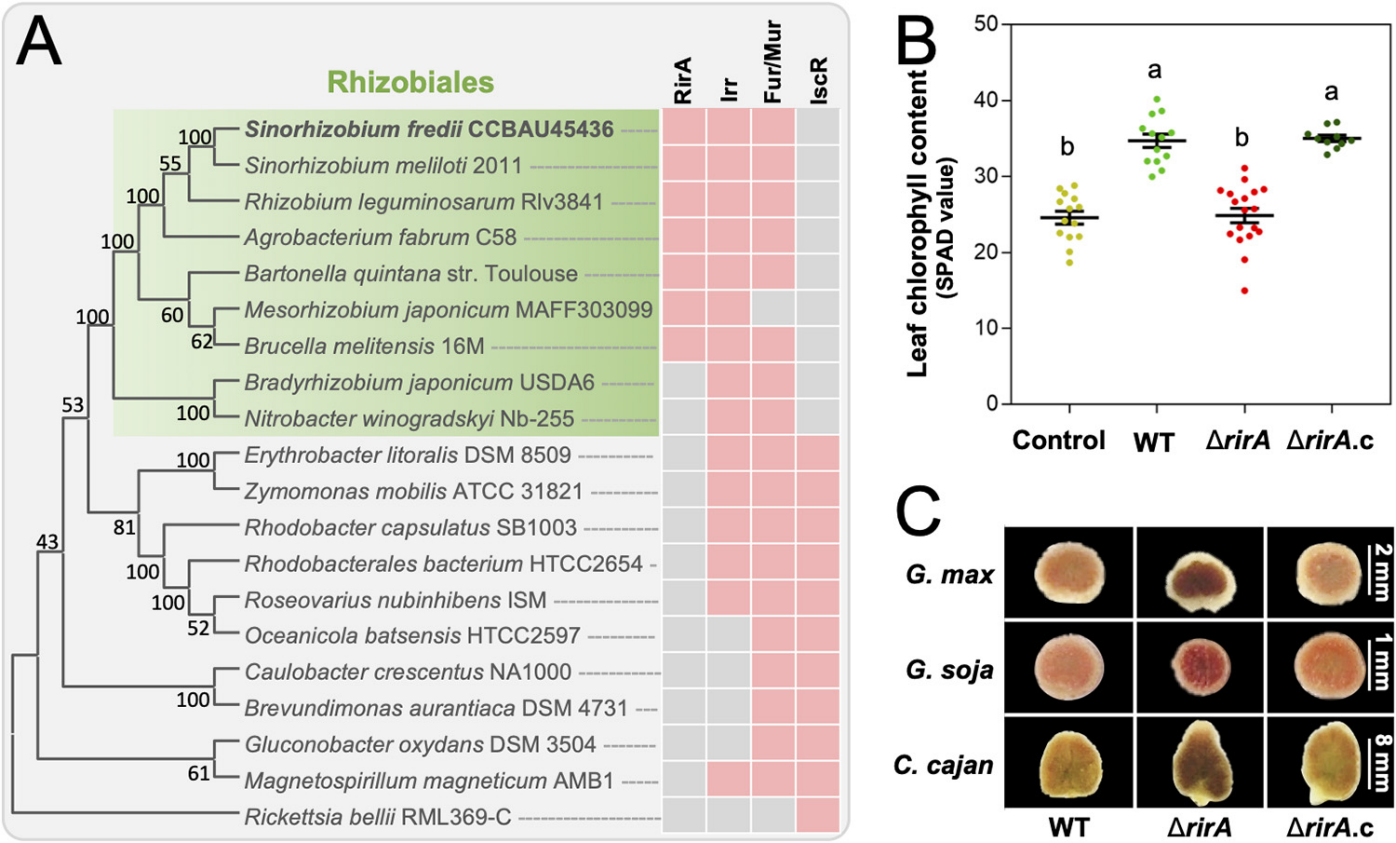
*Rhizobiales*-specific iron regulator RirA supports symbiotic performance of the broad-host-range Sinorhizobium fredii. (A) Phyletic distribution of iron regulators RirA, Irr, Fur (Mur), and IscR in *Alphaproteobacteria*. Pink and gray cells indicate presence and absence, respectively. The neighbor-joining phylogenetic tree of representative species belonging to *Alphaproteobacteria* was constructed based on RpoB. (B) Leaf chlorophyll content of soybean plants inoculated with the Δ*rirA* mutant and its complementary strain Δ*rirA*.c. Different letters indicate significant difference between means (mean ± SE; ANOVA followed by Duncan's test, alpha = 0.05). More than 10 plants from three independent experiments were scored. (C) Vertical section of nodules from Glycine max (soybean), Glycine soja (wild soybean), and Cajanus cajan (pigeon pea) plants inoculated with the Δ*rirA* mutant and its complementary strain. Detailed statistics of symbiotic performance for test strains are shown in Table S1B in the supplemental material.

The importance of iron homeostasis for both eukaryotes and their associated microbiota has been highlighted by anemia caused by iron deficiency (11), local restriction of iron availability as part of the innate immune response in pathogen infection (12), and stress adaptation (13). The iron homeostasis is regulated by Fur in most prokaryotes such as bacilli, cyanobacteria, and *Beta*- and *Gammaproteobacteria* (14). Notably, the Fur-like homologs evolved into a regulator of the manganese uptake (Mur) in *Rhizobiales*, which have Irr and RirA as major iron-responsive regulators (15–17). The *Rhizobiales* order contains many bacteria closely interacting with eukaryote hosts, such as pathogens in *Agrobacterium*, Brucella, and *Bartonella* and legume microsymbionts belonging to *Bradyrhizobium*, *Rhizobium*, *Sinorhizobium*, and *Mesorhizobium* (16). The iron metabolism is crucial for nitrogen fixation activity of rhizobia living intracellularly in legume nodule cells (18). For example, nitrogenase contains the iron protein with a [4Fe-4S] cluster, the molybdenum-iron protein with an [8Fe-7S] cluster, and the FeMo cofactor (MoFe_7_S_9_·homocitrate); the heme-containing leghemoglobin mediates high oxygen flux at low concentrations to avoid inactivation of nitrogenase while maintaining essential respiration processes (19). Irr is present and active under low-iron conditions, while it does not function under iron-replete conditions by either heme-mediated degradation or dissociation from DNA in a species-dependent manner (20–25). RirA was first reported in Rhizobium leguminosarum in 2002 (26), and its orthologs are specific to *Rhizobiales* members belonging to *Rhizobiaceae*, *Mesorhizobiaceae*, *Brucellaceae*, and *Bartonellaceae*, but are not found in *Bradyrhizobiaceae* (15, 16) (Fig. 1A). RirA, together with the Fe-S synthesis regulator IscR (27) and the NO sensor NsrR (28), belongs to the protein family Rrf2, though IscR orthologs are not found in *Rhizobiales* (15) (Fig. 1A). Direct evidence for RirA-DNA binding was just recently provided (29) for the promoter of *fhuA* encoding a putative outer membrane receptor for siderophore vicibactin in R. leguminosarum (30). RirA harboring a [4Fe-4S] cluster directly represses *fhuA* under iron-replete conditions and the conversion into the [2Fe-2S] form and apo-RirA under low-iron conditions lead to impairment and loss of DNA binding ability, respectively (29, 31, 32). Transcriptomic and/or phenotypic characterizations of the *rirA* mutant have demonstrated RirA as an iron-responsive regulator under free-living conditions in R. leguminosarum (26, 32), Sinorhizobium meliloti (33–35), Sinorhizobium fredii (36), and Agrobacterium tumefaciens (25, 37). RirA is required for pathogenesis of A. tumefaciens on tobacco leaves (37, 38). Symbiotic defects were not observed for the *rirA* mutants of R. leguminosarum (26) and S. meliloti (33), both of which undergo irreversible terminal differentiation (impaired reproductive ability) in nodule cells of pea and alfalfa, respectively (39, 40). In contrast, the *rirA* mutant of S. fredii HH103 had impaired symbiotic nitrogen fixation in soybean nodules (36), in which rhizobia still maintain reproduction potential after nodule senescence (40). Collectively, the working mechanisms of *Rhizobiales*-specific RirA in various pathogenic and mutualistic interactions between bacteria and eukaryote hosts remain largely unexplored.

10.1128/mBio.02900-21.6 TABLE S1 Symbiotic performance of Sinorhizobium fredii CCBAU45436 and its derivatives. Download Table S1, XLSX file, 0.02 MB. Copyright © 2022 Liu et al. 2022 Liu et al. https://creativecommons.org/licenses/by/4.0/ This content is distributed under the terms of the Creative Commons Attribution 4.0 International license.

In this work, we focused on RirA from a broad-host-range rhizobium S. fredii CCBAU45436 (SF4) which can form nitrogen-fixing nodules with diverse legumes such as cultivated soybean (Glycine max), wild soybean (Glycine soja), and pigeon pea (Cajanus cajan) (41). The in-frame deletion mutant of *rirA*, rather than *irr*, exhibited severe symbiotic defects, which were characterized by iron overload in nodules, poor intracellular persistence, and impaired symbiotic nitrogen fixation of rhizobia. By screening suppressor mutations and combining chromatin immunoprecipitation (ChIP), reverse genetics, cytological and physiological assays, and evolutionary analysis, we revealed that to maintain iron homeostasis in mutualistic nitrogen-fixing nodules, *Rhizobiales*-specific RirA has been recruited to repress a horizontally transferred gene cluster directing petrobactin siderophore biosynthesis and transport. An evolutionary model regarding the natural assembly of this circuit for siderophore biosynthesis and transport, and its integration with the indigenous regulation cascade, was proposed and discussed.

## RESULTS AND DISCUSSION

### *Rhizobiales*-specific RirA is essential for the symbiotic efficiency of the broad-host-range Sinorhizobium fredii.

Cumulative evidence suggests that Irr binds DNA targets under low-iron conditions (16), while RirA functions under high-iron conditions (29, 31, 33–35). Given the high level of iron-containing nitrogenase and leghemoglobin in nodules (19, 42, 43), nitrogen-fixing rhizobia in nodule cells are supposed to be under iron-replete conditions (18). Then, it could be hypothesized that rhizobial RirA, rather than Irr, might be active in nodules. To test this hypothesis, in-frame deletion mutants Δ*irr*, Δ*rirA*, and Δ*rirA irr* (double mutant) of S. fredii CCBAU45436 (SF4) were compared for their symbiotic performance on wild soybean plants (G. soja) (Table S1A in the supplemental material). The Δ*rirA* and the double mutant Δ*rirA irr* showed a significant reduction in symbiotic efficiency regarding leaf chlorophyll content of host plants (analysis of variance [ANOVA] followed by Duncan's test, alpha = 0.05), while the Δ*irr* and the complementary strain Δ*rirA.c* were indistinguishable from the wild-type SF4 (WT here). The essential role of RirA for symbiotic efficiency was also found on cultivated soybean (G. max) (Fig. 1B) and pigeon pea plants (C. cajan) (Table S1B), which are important legume crops (44, 45). Therefore, symbiotic defects of the Δ*rirA* mutant of the broad-host-range strain SF4 were not dependent on the contrasting development characteristics of spherical determinate (soybean, with transiently active nodule meristems) and elongated indeterminate (pigeon pea, with persistent nodule meristems) nodules (46).

### The *rirA* deletion leads to iron overload in nodules.

**FIG 2:**
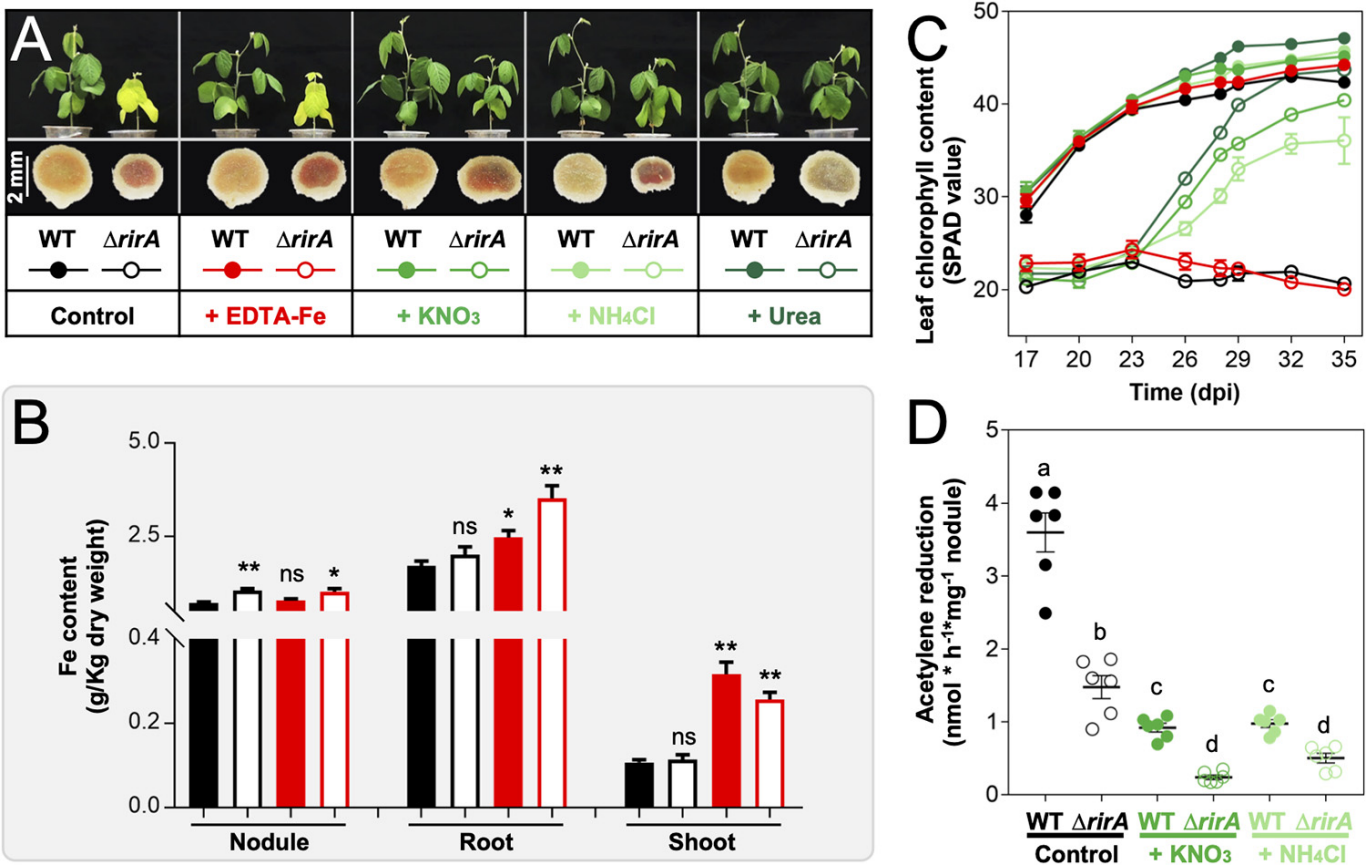
RirA is required for avoiding iron overload in nodules. (A) Growth defects of soybean plants inoculated with the Δ*rirA* mutant can be rescued by supplying chemical nitrogen fertilizers (20 mM KNO_3_, 20 mM NH_4_Cl, or 10 mM urea) but not by iron (1.5 mM EDTA-Fe) at 21 days postinoculation (dpi). Pictures of plants and nodules were taken at 28 dpi. Detailed statistics of symbiotic performance for test strains are shown in Table S1C in the supplemental material. (B) Iron content of nodule, root, and shoot from soybean plants inoculated with the Δ*rirA* mutant, with or without EDTA-Fe supply. Significant difference compared to the treatment of the wild-type strain without EDTA-Fe supply was indicated (*t* test; *, *P* < 0.05; **, *P* < 0.01; ns, nonsignificant; mean ± SE based on samples from three independent experiments). (C) Dynamic changes in leaf chlorophyll content of soybean plants (mean ± SE; more than nine plants from three independent experiments were scored). (D) Symbiotic nitrogen fixation capacity for soybean nodules inoculated with the Δ*rirA* mutant, without or with nitrogen fertilizer supply (20 mM KNO_3_ or 20 mM NH_4_Cl). Different letters indicate significant difference between means based on six biological replicates from two independent experiments (mean ± SE; ANOVA followed by Duncan's test, alpha = 0.05). The same treatments are indicated with the same color scheme across panels A to D.

Notably, both determinate and indeterminate nodules induced by the Δ*rirA* mutant had a characteristic color of darker red or even brown compared to WT and the complementary strain Δ*rirA.c* (Fig. 1C), implying a potential difference in nodule iron content. Then, could the impaired growth of host plants inoculated with the Δ*rirA* mutant be caused by iron overload in nodules and/or biased partition of iron among tissues? To answer this question, 1.5 mM EDTA-Fe was supplied 21 days postinoculation (dpi) of rhizobia, and nitrogen fertilizers (20 mM KNO_3_, 20 mM NH_4_Cl, and 10 mM urea) were added at the same time for comparison (Fig. 2A). Without EDTA-Fe supply, nodules infected by the Δ*rirA* mutant had a significantly higher iron content than those induced by SF4 (Fig. 2B), and the iron content in nodules was not notably increased when EDTA-Fe was supplied. This suggested an iron overload status in nodules infected by the Δ*rirA* mutant. On the other hand, without EDTA-Fe supply, no significant difference in iron content of shoot and root was observed between the treatments of WT and the Δ*rirA* mutant (Fig. 2B). Although the iron content of shoot and root was significantly increased for plants supplied with EDTA-Fe (Fig. 2B, red), dynamic changes in leaf chlorophyll content showed that the growth of soybean plants inoculated with the Δ*rirA* mutant can be rescued by nitrogen fertilizers (green) but not by EDTA-Fe (red) (Fig. 2C, Fig. 2A, and Table S1C). These results suggested that the observed yellow leaves on plants treated with the Δ*rirA* mutant are due to the deficiency of nitrogen supply by rhizobia. Indeed, the symbiotic nitrogen fixation capacity of the Δ*rirA* mutant was significantly lower than WT (Fig. 2D), potentially caused by iron overload mediated by iron uptake systems regulated by RirA (Fig. 2B). In line with this hypothesis, earlier transcriptional analyses of the RirA regulon of R. leguminosarum and S. meliloti under free-living conditions suggest that RirA acts as a repressor for various iron uptake systems under iron-replete conditions (26, 32, 33). Given the existence of strain-specific iron uptake systems among rhizobia (17) and contrasting physiological features and regulation networks between free-living and symbiotic rhizobia (47–49), the RirA regulon of SF4 was further explored in this work.

### Suppressor mutations rescue symbiotic performance of the Δ*rirA* mutant.

**FIG 3:**
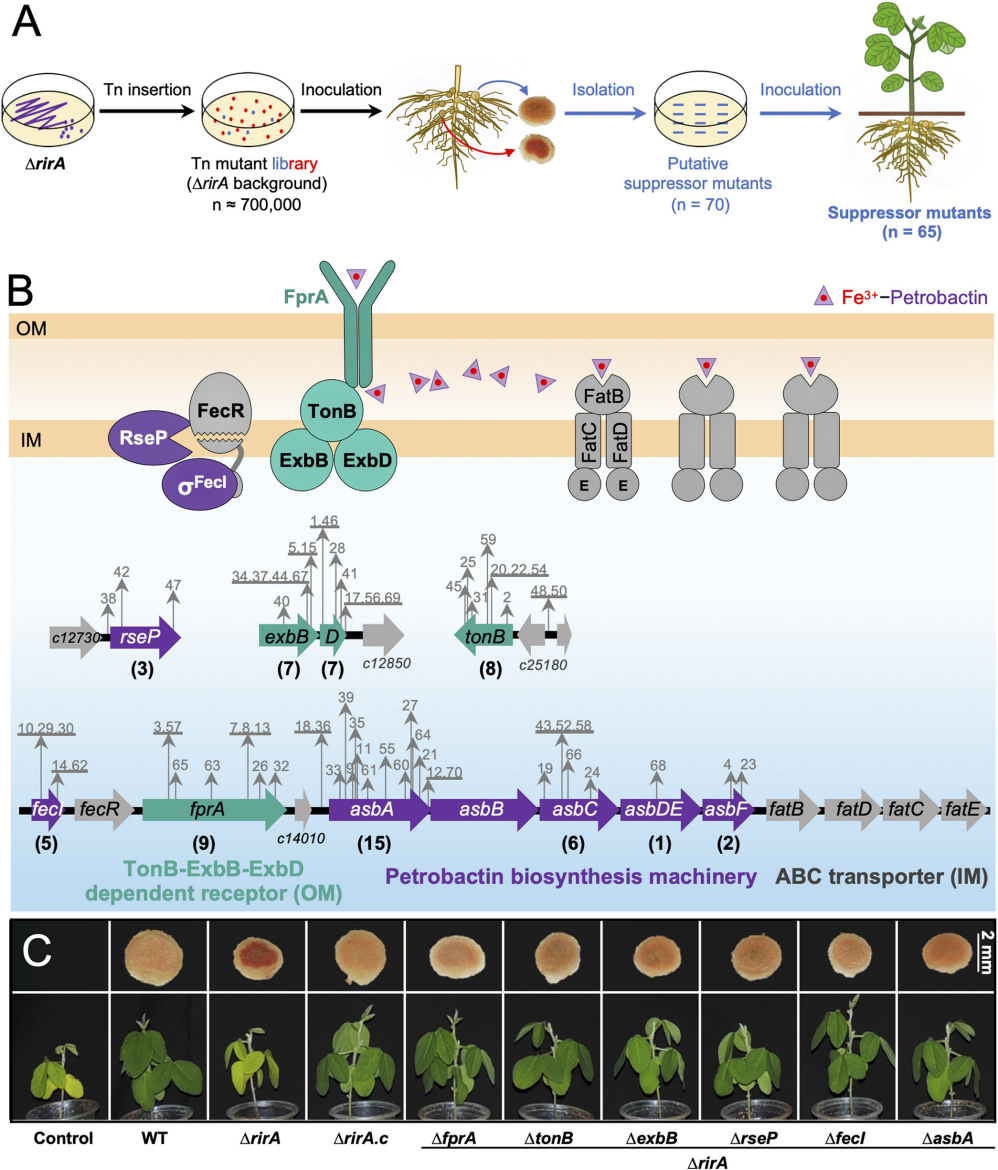
Suppressor mutations rescue symbiotic performance of the Δ*rirA* mutant. (A) Workflow for screening suppressor mutants from a Tn mutant library in the Δ*rirA* background. The number of (putative) suppressor mutants is indicated in brackets. (B) Insertion sites of Tn in the genome of 65 suppressor mutants. Mutant IDs are shown in gray, and the number of independent suppressor mutants identified for individual genes is shown in brackets. (C) Verification of suppressor mutations by reverse genetics in the Δ*rirA* background. The combined mutants were obtained by in-frame deletion of related genes as indicated. Representative pictures of shoot and vertical section of nodules are shown. Detailed statistics of symbiotic performance for test strains are shown in Table S1D, Table S1E, and Fig. S1 in the supplemental material.

Most legume nodules are infected by a single clone despite a large population of rhizobial cells in the rhizosphere such as reported for S. fredii-soybean (50), S. meliloti-alfalfa (51), and Mesorhizobium loti-Lotus japonicum pairs (52). This fishing phenomenon by hosts has been successfully utilized in experimental evolutionary studies screening rhizobial clones compatible with certain soybean cultivars or *Mimosa* plants (53, 54). To screen suppressor mutations which rescue symbiotic defects of the Δ*rirA* mutant, a transposon (Tn) mutant library containing around 700,000 clones was constructed in the Δ*rirA* background (Fig. 3A). A mixture of this library was inoculated on 200 soybean plants, and 70 nodules of color similar to those induced by WT were subject to further isolation, purification, and reinoculation experiments (Fig. 3A). Finally, 65 independent suppressor mutants able to rescue the symbiotic defects of the Δ*rirA* mutant were obtained, and their Tn insertion sites were determined (Fig. 3B). These suppressor mutations were localized in four genomic regions on the chromosome of the multipartite genome of SF4 (48).

10.1128/mBio.02900-21.1 FIG S1 Symbiotic performance of suppressor mutants on wild soybean plants. The combined mutants were obtained by in-frame deletion of related genes in the Δ*rirA* background as indicated. *asbB* and *fatBDCE* were also deleted in the Δ*rirA* background for comparison. Representative pictures of vertical section of nodules are shown. Different letters indicate significant difference between means (mean ± SE; ANOVA followed by Duncan's test, alpha = 0.05) based on more than 13 scored plants from three independent experiments. Detailed statistics of symbiotic performance for test strains are shown in Table S1E. Download FIG S1, PDF file, 0.4 MB. Copyright © 2022 Liu et al. 2022 Liu et al. https://creativecommons.org/licenses/by/4.0/ This content is distributed under the terms of the Creative Commons Attribution 4.0 International license.

Three suppressor mutations were associated with *rseP* (regulator of sigma E, protease) (Fig. 3B) encoding a conserved inner membrane protease. RseP cleaves transmembrane sequences of proteins, including anti-σ^E^ RseA and anti-σ^FecI^ FecR (55, 56), leading to the release of σ^E^ and σ^FecI^ from the cytoplasmic domains of RseA/FecR and the activation of genes involved in extracytoplasmic stress responses and iron uptake genes, respectively, in Gram-negative bacteria (57, 58). Consistent with this model, insertion mutations were found in *fecI* encoding the sigma factor σ^FecI^, but not in its downstream *fecR* encoding the inner membrane-anchored anti-σ^FecI^ (Fig. 3B). Moreover, 20 independent insertion events from 24 suppressor mutants were found in the *asbABCDEF* gene cluster encoding a complete biosynthesis machinery for petrobactin siderophore (catecholate; Fig. 3B) that was first found in an oil-degrading Gram-negative Marinobacter hydrocarbonoclasticus in 2002 (59) and has been intensively studied in the Gram-positive pathogen Bacillus anthracis (59–62). AsbABCDEF of SF4 showed protein identity values ranging from 62% to 69% with those of B. anthracis (strain Sterne) and 58% to 62% with those of M. hydrocarbonoclasticus ATCC 49840. This *asb* gene cluster is, however, rarely found in other rhizobia as detailed below, implying its horizontal transfer among bacteria. It has been reported that extracellular Fe^3+^-petrobactin is recognized by a surface receptor FpuA and then imported into the cytoplasm by multiple redundant ABC transporters in B. anthracis (63). In contrast, Gram-negative bacteria have an outer membrane, and the receptor for Fe^3+^-petrobactin in Gram-negative bacteria is still unknown (3). In this work, there were 6 independent suppressor mutations in *c14000* encoding a TonB-dependent outer membrane siderophore receptor (here named as a putative Fe^3+^-petrobactin receptor FprA) and 13 mutations in genes (*tonB*, *exbB*, and *exbD*) encoding Ton complex components TonB-ExbB-ExbD (Fig. 3B). This is in line with the TonB-ExbB-ExbD-dependent iron transport pathway mediated by various known siderophores and their specific outer membrane receptors in Gram-negative bacteria (3, 64). Within this model, the inner membrane TonB-ExbB-ExbD complex transduces the energy of inner membrane proton motive force to energize the outer membrane transport reactions (64–66).

The rescue effects of these suppressor mutations were further confirmed by the symbiotic performance of combined mutants Δ*rirA fprA*, Δ*rirA tonB*, Δ*rirA exbB*, Δ*rirA rseP*, Δ*rirA fecI*, Δ*rirA asbA*, and Δ*rirA asbB* (in-frame deletion mutants) (Fig. 3C, Fig. S1, and Table S1D and E). Of note, no suppressor mutations were found for any inner membrane ABC transporters in this work, implying multiple redundant inner membrane transporters in SF4 as reported in B. anthracis, which has three inner membrane transporters for Fe^3+^-petrobactin (63). Interestingly, sequence analysis uncovered four genes, just downstream of *asbF*, encoding FatBDCE orthologs, reported as an inner membrane ABC transporter for Fe^3+^-petrobactin in B. anthracis (protein identities range from 56% to 66%) (63). To test if the mutation of *fatBDCE* had a partial suppressor effect in the Δ*rirA* background, the Δ*rirA fatBDCE* in-frame deletion mutant was constructed and inoculated on host plants (Fig. S1). Indeed, its symbiotic performance was higher than that of the Δ*rirA* mutant but lower than the other suppressor mutants (Fig. S1 and Table S1E).

### Suppressor mutations rescue intracellular persistence and nodule iron homeostasis.

**FIG 4:**
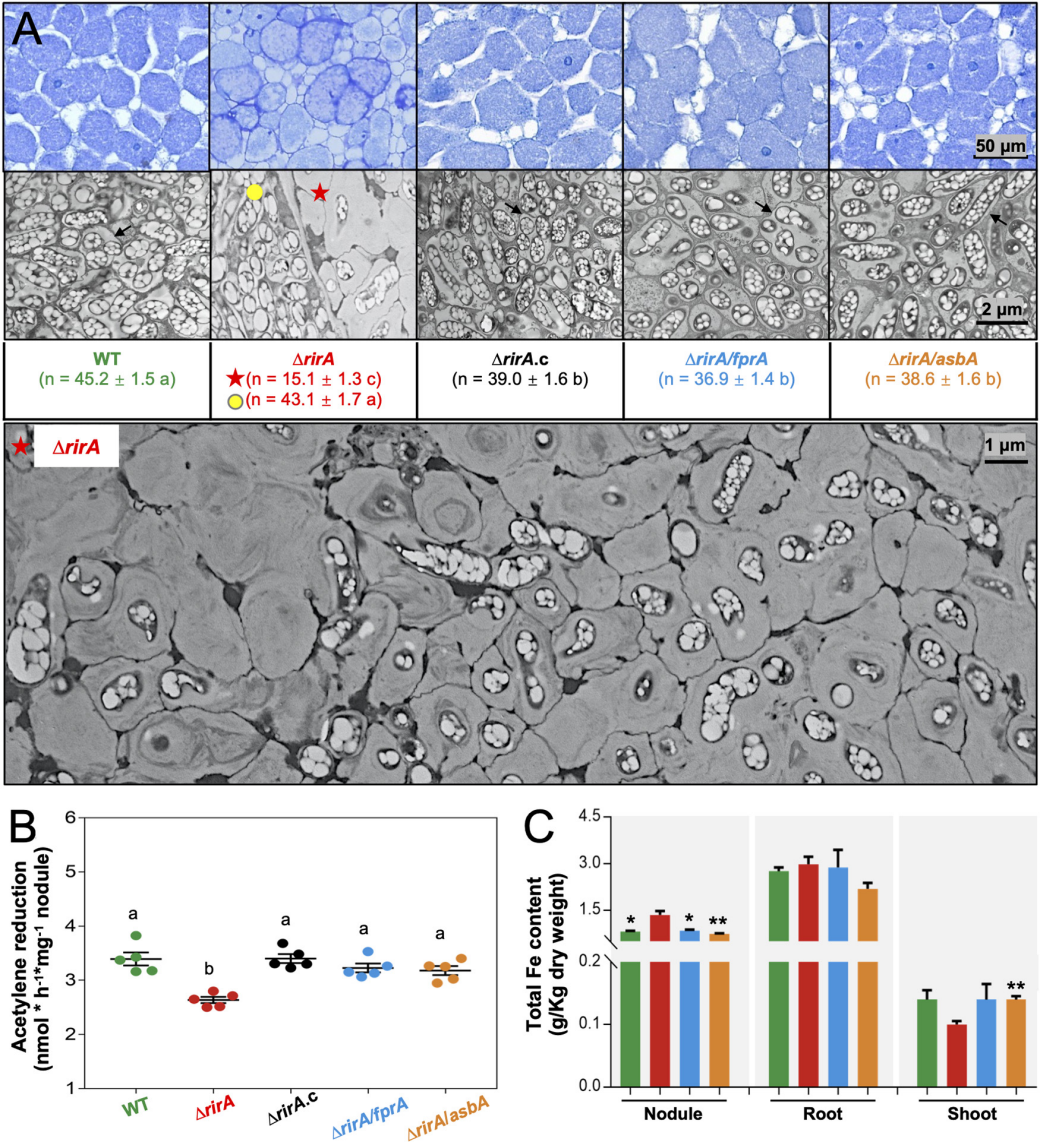
Suppressor mutations rescue defects of the Δ*rirA* mutant in intracellular persistence and nodule iron homeostasis. (A) Thin and ultrathin sections of nodules (28 dpi) observed with light microscopy and transmission electronic microscopy, respectively. The number of bacteroids observed in a 6.35- by 6.35-μm quadrat of infected nodule cells is shown, and significant difference between means is indicated with different letters (mean ± SE of nine quadrats; ANOVA followed by Duncan's test, alpha = 0.05). Filled yellow circle (rare infected nodule cells) and red star (most infected nodule cells) indicate two kinds of nodule cells infected by the Δ*rirA* mutant (see Fig. S2 in the supplemental material for details). Black arrow points to the symbiosome membrane harboring multiple rhizobial cells. (B) Symbiotic nitrogen fixation capacity of soybean nodules (28 dpi). Different letters indicate significant difference between means of five biological replicates (mean ± SE; ANOVA followed by Duncan's test, alpha = 0.05). (C) Iron content of nodule, root, and shoot from soybean plants inoculated with WT and Δ*rirA*, Δ*rirA fprA*, and Δ*rirA asbA* mutants. Significant difference compared to the Δ*rirA* mutant is indicated (*t* test, *, *P* < 0.05; **, *P* < 0.01; mean ± SE based on three independent experiments).

From the suppressor mutations (Fig. 3B), it can be deduced that the dysregulated biosynthesis of petrobactin siderophore (AsbABCDEF) and uptake of Fe^3+^-petrobactin (FprA, etc.) in the Δ*rirA* mutant might cause the observed iron overload in nodules (Fig. 2B) and impair symbiotic nitrogen fixation (Fig. 2D). This hypothesis was further tested with the Δ*rirA asbA* and Δ*rirA fprA* mutants. Soybean nodules (28 dpi) infected by the Δ*rirA* mutant harbored two kinds of infected cells (Fig. 4A and Fig. S2), with rare infected nodule cells harboring bacteria at similar density to that of WT and most infected cells showing a density reduction by 70%, suggesting impaired intracellular persistence but not infection defect for the Δ*rirA* mutant. In contrast, rhizobial density in nodule cells was largely restored for treatments of the Δ*rirA asbA*, Δ*rirA fprA*, and Δ*rirA.c* strains (Fig. 4A). Similarly, symbiotic nitrogen fixation capacity (Fig. 4B) and nodule iron content (Fig. 4C) were restored in the treatments of Δ*rirA asbA* and Δ*rirA fprA* mutants to levels similar to that of WT. These contrasting levels of rhizobial persistence in nodule cells and nodule iron content among treatments are in line with the general negative regulation of iron uptake genes in Gram-negative bacteria under iron-replete conditions (26, 29, 31, 33).

10.1128/mBio.02900-21.2 FIG S2 Most nodule cells infected by the Δ*rirA* mutant harboring low bacterial density. Ultrathin sections of nodules (28 dpi) observed with transmission electronic microscopy. Filled yellow circle (only one in three quadrats) and red star indicate two kinds of nodule cells infected by the Δ*rirA* mutant. Orange stars indicate symbiosomes harboring degrading rhizobia (similar to those in red star cells) in nodule cells of normal bacterial density (yellow circle cells). Download FIG S2, PDF file, 0.6 MB. Copyright © 2022 Liu et al. 2022 Liu et al. https://creativecommons.org/licenses/by/4.0/ This content is distributed under the terms of the Creative Commons Attribution 4.0 International license.

Excess intracellular iron can induce Fenton reactions, which, in turn, lead to cell death via several processes, including ferroptosis, which is characterized by membrane damage (decreased thickness and increased curvature) due to lipid peroxidation (67–70). In line with this recent progress, the Δ*rirA* cells at different degradation stages can be observed in all infected nodule cells (Fig. 4A; the rare nodule cells with normal rhizobial density are shown in Fig. S2), and this degradation process was associated with increased curvature and rupture of symbiosome membranes (Fig. 4A and Fig. S2). These cytological traits were not observed for the Δ*rirA asbA*, Δ*rirA fprA*, Δ*rirA.c*, and WT strains (Fig. 4A). Similarly, under the iron-replete free-living condition (37 μΜ FeCl_3_) (Fig. S3), the generation time of the Δ*rirA* mutant (5.14 ± 0.12 h) was significantly longer than that of the Δ*rirA asbA* (4.55 ± 0.04 h), Δ*rirA fprA* (3.95 ± 0.06 h), Δ*rirA.c* (4.21 ± 0.02 h), and WT (4.19 ± 0.01 h) strains (*t* test, *P* < 0.05). Under the iron-limited condition (0.37 μΜ FeCl_3_), the Δ*rirA* mutant grew at a similar rate as the Δ*rirA fprA*, Δ*rirA.c*, and WT strains (generation time ranged from 5.13 to 5.47 h) while being faster than the Δ*rirA asbA* mutant (6.49 h; *P* < 0.05) (Fig. S3). Therefore, RirA plays an important role in an iron-rich nodule environment to avoid iron overload possibly by directly or indirectly repressing the biosynthesis of petrobactin and uptake of Fe^3+^-petrobactin. This view is supported by the recent finding that DNA binding ability of RirA decreases sequentially from the [4Fe-4S], [2Fe-2S], and apo-RirA forms responding to iron-fluctuating conditions (29, 31, 32).

10.1128/mBio.02900-21.3 FIG S3 Growth curves and generation time (*T*_gen_) for test strains. The iron-replete condition (37 μΜ FeCl_3_) (A) and the iron-deficient condition (0.37 μΜ FeCl_3_) (B) were used. Significant difference compared to the Δ*rirA* mutant is indicated (*t* test, *, *P* value < 0.05; mean ± SE based on three biological replicates). Download FIG S3, PDF file, 0.3 MB. Copyright © 2022 Liu et al. 2022 Liu et al. https://creativecommons.org/licenses/by/4.0/ This content is distributed under the terms of the Creative Commons Attribution 4.0 International license.

### RirA directly represses *fecI*, *tonB*, *exbBD*, and *fprA-asb-fat* operons.

**FIG 5:**
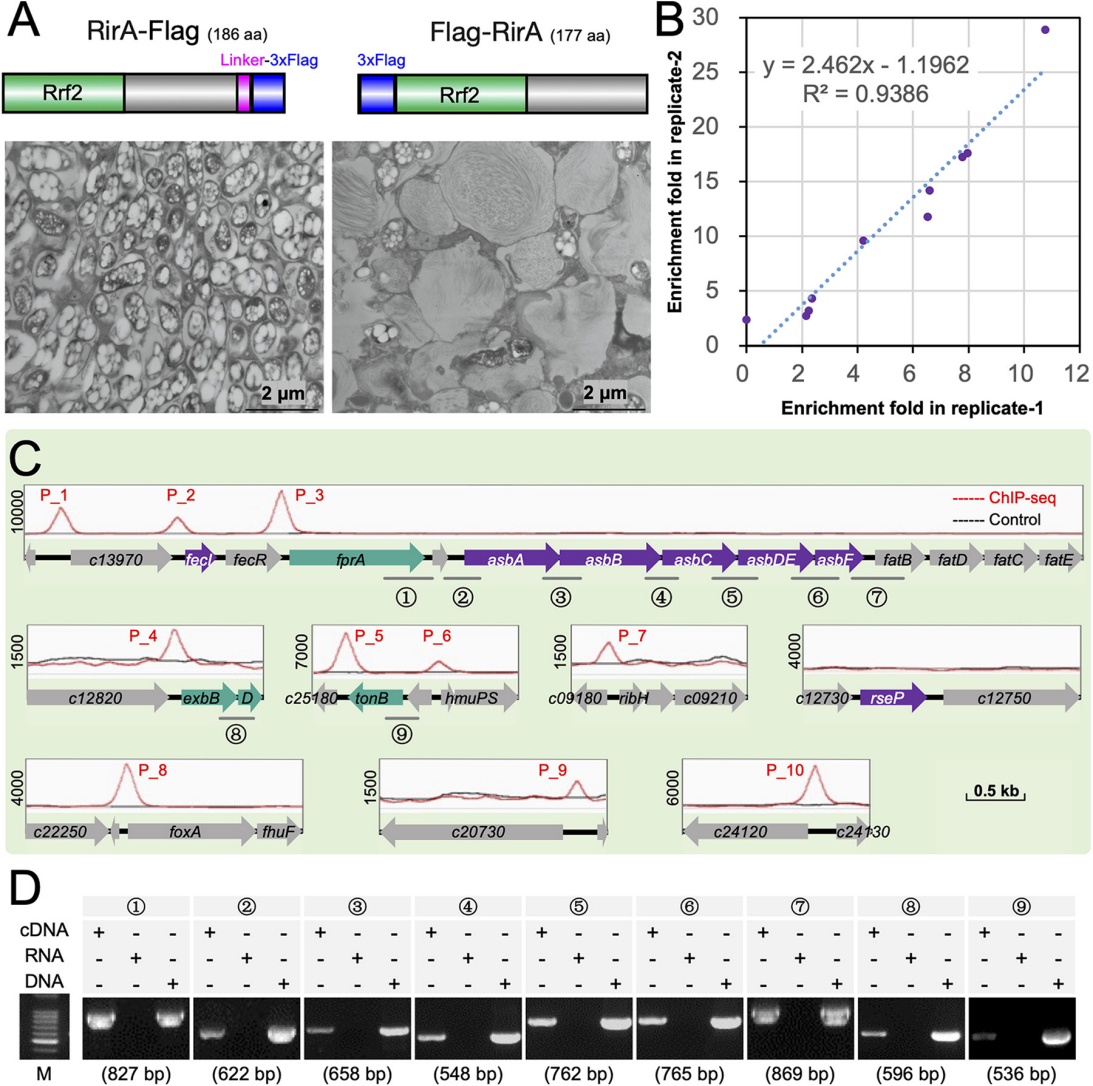
ChIP-seq analysis of RirA targets and determination of related operons by reverse transcriptase PCR. (A) Ultrathin sections of soybean nodules (28 dpi, transmission electronic microscopy) infected by WT with RirA replaced by either RirA-Flag or Flag-RirA. (B and C) Ten binding peaks identified in two independent ChIP-seq analyses of the RirA-Flag strain. (D) Reverse transcriptase PCR reveals three transcripts harboring *fprA-c14010-asbABCDEF-fatB* (primer pairs 1 to 7), *exbB-exbD* (8), and *c25200-tonB* (9) in the Δ*rirA* mutant. The length of fragments targeted by nine pairs of primers is shown in brackets. Log-phase cultures under the iron-replete condition (37 μΜ FeCl_3_) were used in panels B to D.

An earlier computational study of upstream regions of putative iron uptake genes in eight species of *Rhizobiales* harboring RirA revealed a conserved 5′-TGA-(N_9_)-TCA-3′ palindrome motif (15), but a systematic experimental investigation of direct DNA targets has not been done yet. To this end, WT derivatives with RirA replaced by either RirA-Flag or Flag-RirA were constructed (Fig. 5A). The RirA-Flag strain exhibited similar symbiotic performance as the WT, while the N-terminally tagged strain (Flag-RirA) had symbiotic defects, including reduced persistence in nodule cells (Fig. 5A) and leaf chlorophyll content of soybean plants (Table S1B). Consequently, the C-terminally tagged RirA-Flag strain was used in the subsequent ChIP sequencing (ChIP-seq) and ChIP-quantitative PCR (qPCR) experiments.

Two independent ChIP-seq experiments identified 10 significant targets bound by RirA under the iron-replete conditions (37 μΜ FeCl_3_) (Fig. 5B). These binding sites were distributed in seven genomic regions (Fig. 5C and Table S2). Interestingly, 62 out of 65 suppressor mutants had mutations in three regions of this list (Fig. 3B and Fig. 5C), harboring *fecI-fecR-fprA-c14010-asbABCDEF-fatBDCE* (peak 1, peak 2, and peak 3), *exbBD* (peak 4), and *tonB* (peak 5 and peak 6). The other functional genes associated with RirA binding sites include *hmuPSTUV*, encoding a hemin uptake system with the permease HmuU and ATPase HmuV, also involved in utilizing hydroxamate siderophores (including ferrioxamine B and ferrichrome) (peak 6) (71, 72); *foxA-fhuF-fhuP*, encoding an outer membrane receptor, cytoplasmic ferric reductase, and periplasmic component required for hydroxamate siderophore uptake and utilization (peak 8) (71); *c20740-c20750*, encoding putative components of ABC transporter for hydroxamate siderophores (peak 9); *c24120*, encoding a TonB-dependent hemin and ferrichrome siderophore receptor (peak 10); and *ribH*, encoding a dimethyl-8-ribityllumazine synthase (EC 2.5.1.78) involved in riboflavin biosynthesis (peak 7) (73). As the closest binding site of RirA was 513 kb away from the *rirA* coding region (*c03820*), it is clear that RirA is a pathway-specific regulator but not a canonical cluster-situated transcriptional regulator (74, 75) for iron homeostasis.

10.1128/mBio.02900-21.7 TABLE S2 Genes surrounding RirA binding peaks identified in ChIP-seq. Download Table S2, XLSX file, 0.01 MB. Copyright © 2022 Liu et al. 2022 Liu et al. https://creativecommons.org/licenses/by/4.0/ This content is distributed under the terms of the Creative Commons Attribution 4.0 International license.

Further reverse transcriptase PCR analysis under the iron-replete condition (37 μΜ FeCl_3_) (Fig. 5D) revealed that *fprA*-*c14010-asbABCDEF-fatB* was cotranscribed and associated with peak 3. Similarly, *exbB-exbD*, associated with peak 4, and *c25200-tonB*, associated with peak 6, were cotranscribed, respectively (Fig. 5D). These results inspired us to propose a hypothesis that all suppressor mutations except those in the *rseP* gene (Fig. 3B and Fig. 5D) were located in transcribed regions under direct negative regulation by RirA within iron-rich nodule cells.

**FIG 6:**
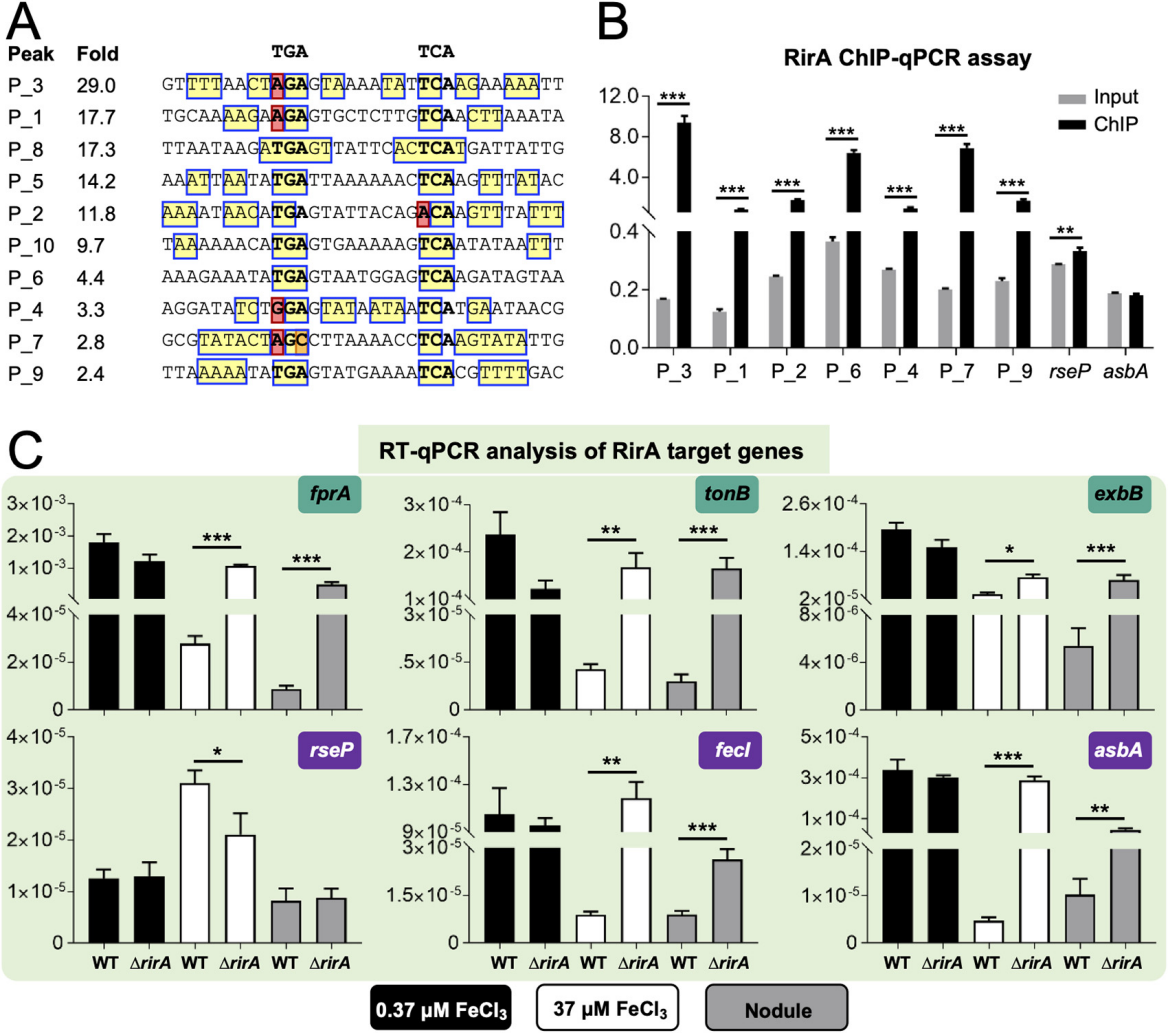
ChIP-qPCR and RT-qPCR analyses of RirA targets. (A) Palindrome present in RirA target sequences. Recruitment levels of RirA (fold) are shown. (B) ChIP-qPCR verification of RirA targets with relatively low RirA recruitment levels in ChIP-seq experiments (P_6, P_4, P_7, and P_9). Negative (upstream sequences of *rseP* or *asbA* genes) and positive (P_3, P_1, and P_2) controls are included for comparison. (C) RT-qPCR analysis of transcriptional profiles of RirA target genes in free-living cultures under iron-replete (37 μM FeCl_3_) or -deficient (0.37 μM FeCl_3_) conditions and rhizobia within soybean nodules (28 dpi). The transcription level relative to the 16S rRNA gene is shown. Significant differences are indicated in panels B and C (mean ± SE based on three independent experiments; *t* test, *, *P* < 0.05; **, *P* < 0.01; ***, *P* < 0.001).

Sequence analysis of RirA binding peaks (Fig. 6A) revealed that 5 out of 10 peaks possessed the predicted 5′-TGA-(N_9_)-TCA-3′ palindrome motif proposed earlier (15), though notable variations in palindrome sequences and RirA recruitment levels can be observed. For those peaks showing relatively low affinity with RirA in ChIP-seq experiments (peak 6, peak 4, peak 7, and peak 9) (Fig. 6A), ChIP-qPCR analysis demonstrated a comparable recruitment level of RirA to these genomic regions and to other positive-control regions (peak 3, peak 1, and peak 2) (Fig. 6B). In contrast, no notable recruitment of RirA to the upstream regions of *rseP* or *asbA* was observed. Therefore, reliable direct targets of RirA were identified (Fig. 5C), and potentially negative or positive regulation of associated operons (Fig. 5D) by RirA can be tested (Fig. 6C and Fig. S4). Within soybean nodules and in the iron-replete medium (37 μΜ FeCl_3_) but not under the iron-deficient condition (0.37 μΜ FeCl_3_), transcriptional levels of *fprA*, *tonB*, *exbB*, *asbA*, and *fecI* were significantly upregulated in the Δ*rirA* mutant compared to those in WT (Fig. 6C). These results suggested that RirA may directly repress the operons, including *fprA*-*c14010-asbABCDEF-fat*, *c25200-tonB*, *exbB-exbD*, and *fecI*, to avoid iron overload in nodules and that suppressor mutations in these operons (Fig. 3B) can rescue intracellular persistence and symbiotic nitrogen fixation of the Δ*rirA* mutant (Fig. 4). Although *foxA*, *hmuP*, *c25180*, and *c24120* associated with peak 8, peak 6, peak 5, and peak 10, respectively, were also upregulated in nodules infected by the Δ*rirA* mutant (Fig. S4), no suppressor mutations were identified in the corresponding genomic regions of the Δ*rirA* mutant (Fig. 3B).

10.1128/mBio.02900-21.4 FIG S4 RT-qPCR analyses of RirA targets. Free-living cultures under iron-replete (37 μM FeCl_3_) or -deficient (0.37 μM FeCl_3_) conditions and rhizobia within soybean nodules (28 dpi). The transcription level relative to 16S rRNA gene is shown. Significant differences are indicated (mean ± SE based on three independent experiments. *t* test, *, *P* < 0.05; **, *P* < 0.01; ***, *P* < 0.001). Download FIG S4, PDF file, 0.3 MB. Copyright © 2022 Liu et al. 2022 Liu et al. https://creativecommons.org/licenses/by/4.0/ This content is distributed under the terms of the Creative Commons Attribution 4.0 International license.

These results implied that upregulation of uptake systems for hemin (*hmuP* and *c24120*) and hydroxamate siderophores, including ferrioxamine B (*foxA*) and ferrichrome (*c24120*) (71), may not be detrimental to persistence of the Δ*rirA* mutant within nodule cells. This view is supported by the fact that the test strain SF4 has no synthetic gene cluster for hydroxamate siderophores, which are, however, present in R. leguminosarum, S. meliloti, and a few Bradyrhizobium japonicum strains (76, 77). This implied that SF4 may use these specific siderophore uptake systems to utilize public ferric hydroxamate siderophores produced by other microbiota members to obtain competitive benefits during its free-living stage in the soil and rhizosphere (3). This is consistent with an earlier report that hydroxamate siderophores were produced by rhizospheric bacterial isolates but not those nodule isolates of C. cajan (78), one host of S. fredii SF4.

### Siderophore production regulated by RirA and the RseP-FecR-σ^FecI^ cascade.

**FIG 7:**
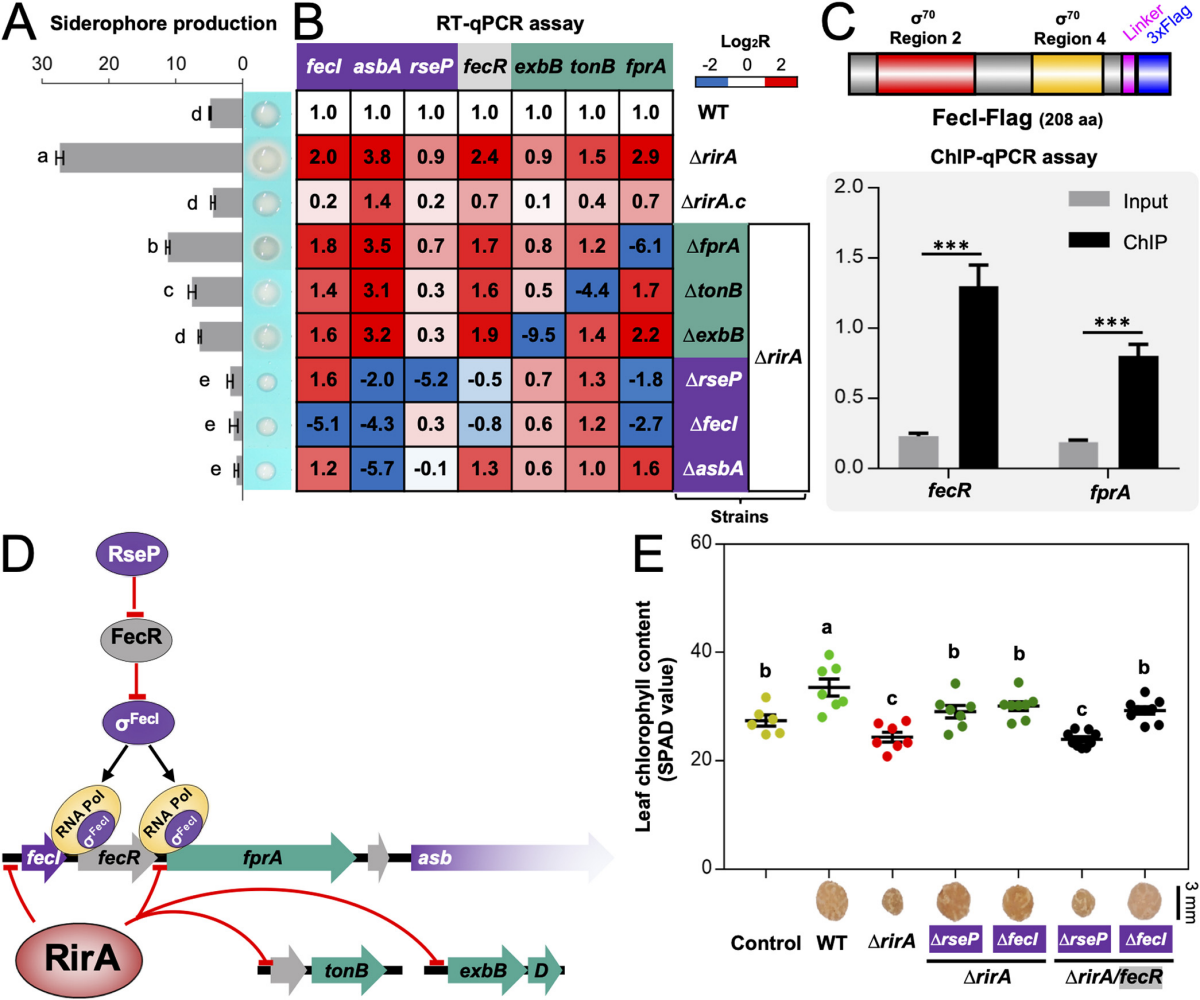
Repression of siderophore production is mainly mediated by direct transcriptional silencing of the sigma factor FecI by RirA. (A) Impaired siderophore production by suppressor mutants compared to the Δ*rirA* mutant. (B) RT-qPCR analysis of genes involved in siderophore production. The transcriptional levels are normalized compared to those in WT. (C) FecI-Flag directly binds the promoter regions of *fecR* and *fprA* revealed by ChIP-qPCR in the Δ*rirA* mutant. Significant enrichment is indicated (mean ± SE based on three biological replicates. *t* test, ***, *P* < 0.001). (D) Working model for repression of the siderophore biosynthesis pathway by RirA. (E) The regulatory role of RseP depends on FecR revealed by symbiotic performance of related combined mutants. Different letters indicate significant difference between means in panels A and E (mean ± SE; ANOVA followed by Duncan's test, alpha = 0.05; three and seven biological replicates in panels A and E, respectively).

In line with direct repression of the operons harboring *fprA*-*c14010-asbABCDEF-fat*, *c25200-tonB*, *exbB-exbD*, and *fecI* by RirA under iron-replete conditions, siderophore overproduction by the Δ*rirA* mutant was largely rescued by suppressor mutations of *fprA*, *tonB*, *exbB*, *fecI*, and *asbA* (Fig. 7A). This suggested that TonB-ExbB-ExbD and the putative TonB-dependent ferric petrobactin receptor FprA were also involved in regulating siderophore production. This regulation can be at the posttranscriptional level as revealed by reverse transcriptase quantitative PCR (RT-qPCR) (Fig. 7B): *asbA* was just slightly downregulated in the Δ*rirA fprA*, Δ*rirA tonB*, and Δ*rirA exbB* mutants compared to the Δ*rirA* mutant. In contrast, the double mutants Δ*rirA fecI* and Δ*rirA rseP* showed a significant downregulation of *asbA* and *fprA* (Fig. 7B) and had nearly undetectable siderophore production, which was similar to the Δ*rirA asbA* mutant (Fig. 7A). This implied that the inner membrane protease RseP and σ^FecI^ were involved in activating the operon harboring *fprA*-*c14010-asbABCDEF-fat*.

Indeed, σ^FecI^ can directly bind the upstream DNA of *fprA* as shown in ChIP-qPCR assay using the *ΔrirA* mutant carrying FecI-Flag (Fig. 7C) (Flag-FecI was not functional, as shown in Fig. S5). It has been demonstrated that RseP homologs have a conserved function of cleaving transmembrane sequences of anti-σ factors, including anti-σ^FecI^ FecR (55, 56), leading to the release of σ^FecI^ (57, 58). This RseP-FecR-σ^FecI^ regulatory cascade is consistent with that suppressor mutations, rescuing defects of the *ΔrirA* mutant, were found in *rseP* and *fecI* but not in *fecR* (Fig. 3B). Notably, *fecR*, but not *fecI*, was downregulated in the *ΔrirA rseP* mutant (Fig. 7B), suggesting that release of σ^FecI^ from FecR is required for activating *fecR* transcription. This view was supported by the downregulation of *fecR* in the *ΔrirA fecI* mutant (Fig. 7B) and direct binding of *fecR* upstream DNA by σ^FecI^ (Fig. 7C). Therefore, the upregulation of *fprA*-*c14010-asbABCDEF-fat* and *fecR*, but not *c25200-tonB*, *exbB-exbD*, and *fecI* in the *ΔrirA* mutant depended on the RseP-FecR-σ^FecI^ cascade (Fig. 7D). Consistent with this scenario, the suppressing effect of the *rseP* mutation in the *ΔrirA* mutant required FecR since the *ΔrirA rseP fecR* triple mutant exhibited similar symbiotic defects as the *ΔrirA* mutant regarding the leaf chlorophyll content of soybean plants (Fig. 7E). Moreover, the *ΔrirA fecI fecR* triple mutant was undistinguishable from the *ΔrirA fecI* double mutant in symbiotic performance (Fig. 7E).

10.1128/mBio.02900-21.5 FIG S5 Symbiotic performance of the Δ*rirA* mutant carrying either the nonfunctional Flag-FecI or functional FecI-Flag. Different letters indicate significant difference between means (mean ± SE; ANOVA followed by Duncan's test, alpha = 0.05). Download FIG S5, PDF file, 0.8 MB. Copyright © 2022 Liu et al. 2022 Liu et al. https://creativecommons.org/licenses/by/4.0/ This content is distributed under the terms of the Creative Commons Attribution 4.0 International license.

Taken together, these results uncovered a hierarchical regulatory cascade modulating iron homeostasis of rhizobia (Fig. 7D). It involves a pathway-specific regulator, RirA, acting at the first level and RseP-FecR-σ^FecI^ acting at the second level. Within legume nodules or other iron-replete conditions, *Rhizobiales*-specific RirA directly represses transcription of σ^FecI^, the TonB-ExbB-ExbD complex, the petrobactin biosynthesis machinery Asb, the putative outer membrane petrobactin receptor FprA, and one of the ABC transporters for petrobactin (Fat). Without a functional RirA, σ^FecI^ is involved in direct transcriptional activation of Asb, FprA, and Fat, and this process requires cleaving anti-σ^FecI^ FecR by the inner membrane protease RseP.

### Assembly and integration of horizontally transferred *fprA-asb-fat* in *Rhizobiales*.

**FIG 8:**
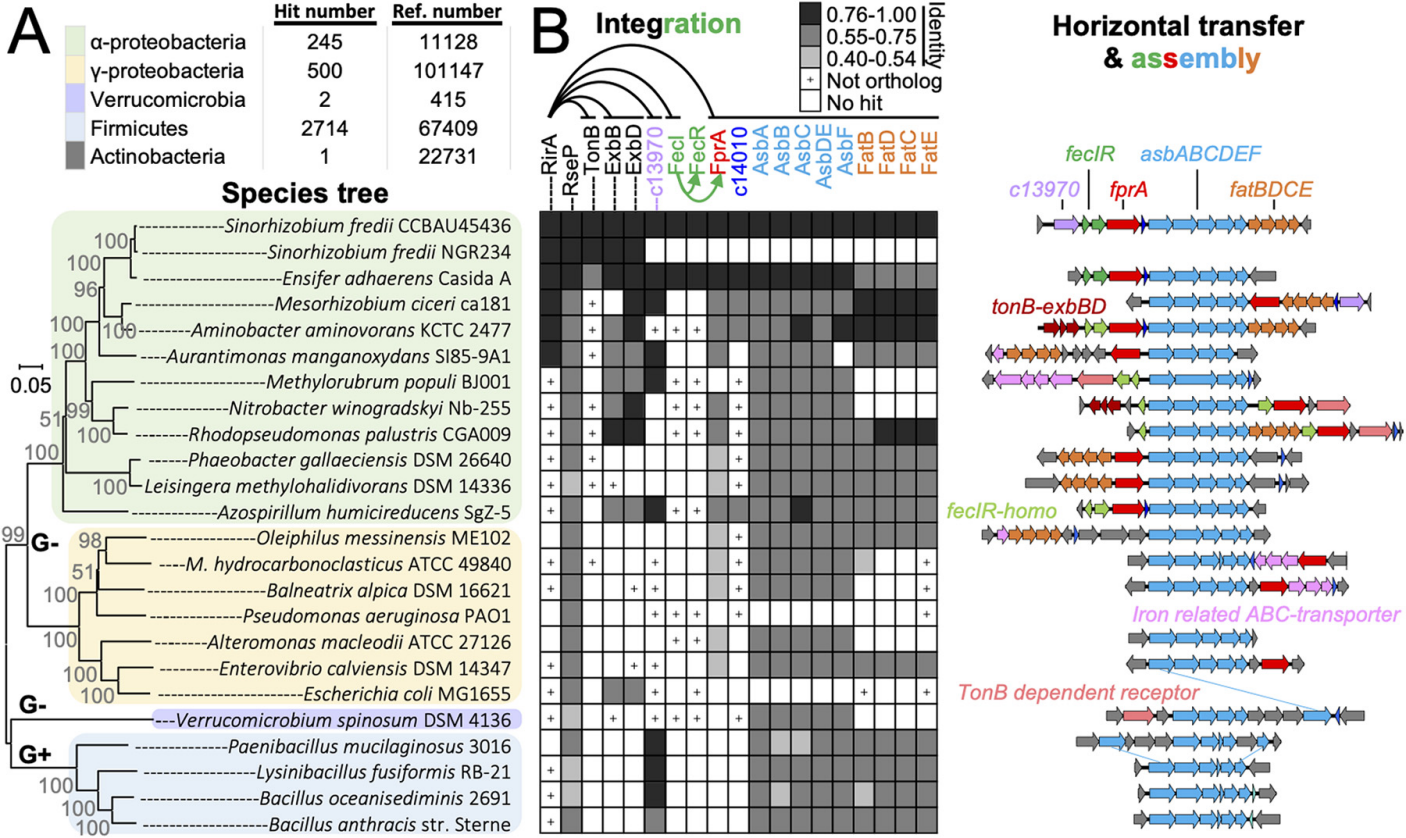
Assembly and integration of horizontally transferred *fprA-asb-fat* in *Rhizobiales*. (A) The number of genomes harboring AsbABCDEF orthologs and the number of reference genomes available in the NCBI Genome resource are shown. The neighbor-joining phylogenetic tree of representative species belonging to different phyla/classes was constructed based on RpoB (G+ and G−, Gram-positive and Gram-negative bacteria, respectively). (B) Phyletic distribution (left) and synteny analysis (right) of genes involved in petrobactin synthesis (*asb*) and transport (*fat* and *fprA*). +, presence of bidirectional best hit, but not ortholog, as revealed by phylogenetic analyses. Protein identity values are indicated by grayscale of three grades.

Sequence analysis revealed that orthologous petrobactin biosynthesis machinery AsbABCDEF (60) can be sporadically found in 3,462 bacterial genomes, with 2,714 in *Firmicutes* (2,626 from 19 species of *Bacillus*), 500 in *Gammaproteobacteria* (173 from 6 species of Pseudomonas), 245 in *Alphaproteobacteria* (49 from 2 species of *Ensifer* and 21 from 2 species of *Sinorhizobium*), 2 in *Verrucomicrobia*, and 1 in *Actinobacteria*. The high protein identity values between AsbABCDEF of S. fredii CCBAU45436 and those of Gram-positive (52% to 69%) or Gram-negative bacteria (55% to 93%) (Fig. 8A and B) imply the horizontal transfer of accessory petrobactin biosynthesis gene cluster *asb* among bacteria. FatBDCE is one of the known petrobactin ABC transporters in B. anthracis (63) and had a similar phyletic distribution pattern as AsbABCDEF in both Gram-positive and Gram-negative bacteria (Fig. 8B). The outer membrane receptor FprA had an even higher Asb co-occurrence frequency than FatBDCE in Gram-negative bacteria (Fig. 8B), in line with current knowledge that different siderophores are recognized by specific outer membrane receptors in Gram-negative bacteria (3) and multiple ABC transporters can be recruited for transporting petrobactin into the cytoplasm as demonstrated in B. anthracis (63). It is noteworthy that the co-occurrence of *fprA*, *fatBDCE*, and *asbABCDEF* within a single genomic locus can be found in distant-related species belonging to *Alphaproteobacteria* (Fig. 8B), implying horizontal transfer events. The *fprA-c14010-asbABCDEF-fatBDCE* cluster of S. fredii CCBAU45436 (belonging to *Rhizobiaceae*) was found in the same synteny in Aminobacter aminovorans (belonging to *Phyllobacteriaceae*) (Fig. 8B). FatBDCE of S. fredii CCBAU45436 was more similar to those of A. aminovorans KCTC 2477 (79% to 85%) than to those of Ensifer adhaerens Casida A (61% to 70%) that is closer to S. fredii in species tree (79). However, FprA, c14010 and AsbABCDEF of S. fredii CCBAU45436 were closer to those orthologs of E. adhaerens Casida A (85% to 93%) than to those of A. aminovorans KCTC 2477 (67% to 80%). This suggested that two gene clusters, *fatBDCE* and *fprA-c14010-asbABCDEF*, from different donors were integrated into the same locus in S. fredii CCBAU45436. Although homologs of FecIR were encoded within the same syntenic locus with *fprA-c14010-asbABCDEF* in these three species (Fig. 8B), FecIR of S. fredii CCBAU45436 had protein identity values of 81% to 89% and 41% to 45% with those from E. adhaerens Casida A and *A.* aminovorans KCTC 2477, respectively.

### Conclusions.

The data obtained in this work support a naturally “synthetic” model that the petrobactin biosynthesis (*asbABCDEF*) and (inner) membrane transporter genes (*fatBDCE*) can be horizontally transferred among Gram-positive and Gram-negative bacteria, and within Gram-negative bacteria, the outer membrane receptor gene *fprA* was recruited to form the biosynthesis and transporter gene cluster that can be transferred and integrated into various chassis Gram-negative bacteria in nature. In the broad-host-range rhizobium S. fredii CCBAU45436, *Rhizobiales*-specific RirA and the indigenous RseP-FecR-σ^FecI^ constitute a two-layer cascade to modulate transcription of the foreign *fprA-c14010-asb-fat* polycistron directing biosynthesis and transport of the petrobactin siderophore. Moreover, RirA also directly regulates indigenous operons encoding the TonB-ExbB-ExbD motor complex that supports the outer membrane transport reactions. Particularly in mutualistic nitrogen-fixing legume nodules, available evidence supports a working model in which this naturally synthetic circuit for petrobactin biosynthesis and transport should be transcriptionally shut down by RirA to avoid iron overload and intracellular persistent deficiency. Given the global importance of the public goods iron and ferric siderophores (2, 3, 80) and the high frequency of horizontal gene transfer in the biosphere (81), the assembly and integration model uncovered in this work can also improve our evolutionary understanding of siderophore biology and ecology in pathogenic interactions and microbiome study of various niches.

## MATERIALS AND METHODS

### Bacterial strains, plasmids, and growth conditions.

Bacterial strains and plasmids used in this study are listed in Table S3 in the supplemental material. *Sinorhizobium* strains were grown at 28°C in tryptone-yeast extract (TY) medium (82), YEM medium (82), or modified VMM medium (83). Escherichia coli strains were cultured in Luria-Bertani (LB) medium at 37°C. The antibiotic concentrations were 50 μg/mL of kanamycin (Km), 10 μg/mL of trimethoprim (Tmp), 30 μg/mL of gentamicin (Gen), and 10 μg/mL of tetracycline (Tc). To determine the growth of rhizobia, the mid-log-phase culture in TY medium was washed once and resuspended in physiological saline to an optical density at 600 nm (OD_600_) equivalent to 0.6, and the suspension was inoculated to VMM medium containing 0.37 μM or 37 μM FeCl_3_ with an initial OD_600_ equivalent of 0.02. Growth curves were monitored by the Bioscreen C (Oy Growth Curves Ab Ltd.).

10.1128/mBio.02900-21.8 TABLE S3 Strains and plasmids used in this study. Download Table S3, XLSX file, 0.01 MB. Copyright © 2022 Liu et al. 2022 Liu et al. https://creativecommons.org/licenses/by/4.0/ This content is distributed under the terms of the Creative Commons Attribution 4.0 International license.

### Construction of plasmids and reverse genetic manipulations.

Primers used for DNA amplification are listed in Table S4. Genomic DNA of S. fredii CCBAU45436 and a pUC57 derivative carrying a chemically synthesized coding sequence for GGGS-GGGGS flexible linker and 3×FLAG tag were used as the templates for DNA amplification. All in-frame deletion mutants were constructed by homologous recombination using the plasmid pJQ200SK (84) that does not replicate in S. fredii (40). The upstream and downstream fragments flanking the target regions were amplified by high-fidelity PCR using primers carrying ∼17- to 20-nucleotide (nt) homologous arms for seamless assembly cloning. The resultant homologous fragments were then assembled into the linearized pJQ200SK (digested by SmaI) by using commercial kit (Tiangen). The derived plasmids harbored by positive clones were verified using PCR and Sanger sequencing and then conjugated into S. fredii strains with the helper plasmid pRK2013 (85). Single-crossover clones were screened for resistance to gentamicin and further subject to passage cultivation and counterselection for double recombinants on TY agar plates added with 5% sucrose. Double-crossover clones were verified by colony PCR and Sanger sequencing. The mutants Δ*rirA*, Δ*rirA fecI*, and Δ*rirA rseP* were used as recipient strains to construct related double and/or triple mutants. To generate the *in situ rirA* complementary strain, the pJQ200SK-derived plasmid carrying the full-length *rirA* coding sequence and up-/downstream fragments was constructed, and the same procedures as in the deletion experiment were carried out to obtain the expected strain. Similarly, the knock-in of 3×FLAG-tag coding sequence into the N/C termini of *rirA* or *fecI* was also accomplished by homologous recombination using the pJQ200SK-derived plasmids (Table S3).

10.1128/mBio.02900-21.9 TABLE S4 Primers used in this study. Download Table S4, XLSX file, 0.02 MB. Copyright © 2022 Liu et al. 2022 Liu et al. https://creativecommons.org/licenses/by/4.0/ This content is distributed under the terms of the Creative Commons Attribution 4.0 International license.

### Tn insertion library construction and suppressor screen.

To construct the Tn insertion library, pSAM_Bt carrying *mariner* himar1C9 transposase (86) was modified into pSAM_Sf by using the P*rpoD* promoter from S. fredii CCBAU45436 to drive the expression of transposase (Table S3). The pSAM_Sf was conjugated into the Δ*rirA* mutant by triparental conjugation with the helper plasmid pRK2013. Approximately 700,000 colonies formed by Tn mutants were collected and mixed in a resuspension (physiological saline; OD_600_ of 0.5). This suspension was inoculated to 200 seedlings of cultivated soybean to screen Tn mutants carrying suppressor mutations. Briefly, nodules with section color similar to those induced by wild type were subject to further isolation and purification, and all isolates were reinoculated on cultivated and wild soybean plants to verify their symbiotic performance. To identify the Tn insertion sites, an adapter ligation-mediated PCR method was employed. Briefly, the purified genomic DNA of each isolated strain was digested by restriction enzyme MmeI and subsequently treated with calf intestinal alkaline phosphatase (CIP). The ligation product of the digested genomic DNA fragment with oligonucleotide adapter annealed from LIB_AdaptT and LIB_AdaptB′ served as the template for PCR amplification by using primers Tn5_L and LIB_PCR_3. The Tn flanking sequences were identified by Sanger sequencing.

### Plant assays, nitrogenase activity assays, and cytological microscopy.

All seeds were surface sterilized in 3% NaClO (wt/vol) solution and germinated for ∼36 to 48 h in the dark as described previously (54). Seedlings were inoculated with 1 mL physiological saline suspension of rhizobia with fan OD_600_ of 0.2. Plants were grown in vermiculite moistened with low-N nutrient solution in Leonard jars at 24°C with day/night cycles of 12:12 h. When required, EDTA-Fe, KNO_3_, NH_4_Cl, and urea were added at the bottom to the desired concentration. Leaf chlorophyll content, nodule number, and shoot dry weight were determined at 28 days postinoculation (dpi) or at the indicated sampling time as described previously (87). Nitrogenase activity of intact nodules was performed using the acetylene reduction method at 28 dpi (88). All plant assays were performed in at least two independent experiments.

To determine the iron content of plant tissues, samples of shoots, roots, and nodules of soybean plants were collected at 28 dpi and dried at 70°C to a constant weight. Samples were ground into a powder that could pass through a 0.5-mm sieve. Samples of 0.1 to 0.3 g were digested overnight with 8 mL HNO_3_ (65% to 68%) by microwave using CEM MARS 6 (CEM). The resultant samples were further digested at 160°C until the volume reached 2 mL. The cooled samples were diluted with water to 25 mL. After standing for 20 min, 10 mL supernatant was loaded onto the inductively coupled plasma-atomic emission spectrometer (ICP-AES; Thermo Fisher Scientific; iCAP 6300) to determine the iron content. The iron content values were standardized using the dry weight of samples. Three independent experiments were performed.

For cytological observation, nodules harvested at 28 dpi were fixed with 2.5% (vol/vol) glutaraldehyde in 0.05 M cacodylate buffer before preparing semithin and ultrathin sections as described earlier (89). Semithin sections were observed under an Olympus BX53F light microscope after staining with 0.01% toluidine blue in 0.1 M phosphate buffer at pH 7.2, while ultrathin sections were observed in a JEM-1230 transmission electron microscope (TEM).

### Detection of siderophore production.

Siderophore production was determined by a universal chemical assay using chrome azurol S (CAS) (90). Briefly, 5 μL mid-log-phase rhizobial culture resuspended in physiological saline at an OD_600_ of 0.2 was dropped on a YEM agar plate containing 10% volume CAS solution. After 3 days, the appearance of an orange halo on CAS agar plates indicates siderophore production. To quantify siderophore production in liquid culture, the supernatant of S. fredii cultures (OD_600_ of 3.0) grown in YEM was mixed 1:1 with the CAS assay solution, and the absorbance at 630 nm (Ar) was measured after incubation for 5 min at room temperature. The absorbance of a 1:1 mixture of fresh YEM medium and CAS solution was also determined and recorded as. The siderophore units were calculated by the following formula: [(Ar − As)/Ar] × 100 = % siderophore units.

### RNA extraction, RT-qPCR, and RT-PCR.

Rhizobial cultures in VMM medium containing 0.37 or 37 μM FeCl_3_ were collected at an OD_590_ of 0.6. Total RNA from these free-living cells was extracted by using an RNAprep pure cell/bacteria kit (Tiangen). Extraction of total RNA of both host and bacteroid in nodules was performed as described previously (48). cDNA was synthesized by using a FastKing RT kit with gDNase (Tiangen). RT-qPCR was performed by using SYBR Green real-time PCR mix (GenStar) and an ABI QuantStudio 6 Flex System real-time PCR system. The 16S rRNA gene was used as an internal control to normalize the relative transcription levels of target genes. Three biological replicates were performed. To determine the cotranscription of adjacent genes, reverse transcription-PCR (RT-PCR) was conducted by using the 2×Taq PCR StarMix (GenStar). cDNA synthesized from total RNA sample extracted from the Δ*rirA* culture grown in VMM medium containing 37 μΜ FeCl_3_ was used as the template. Primers used in RT-PCR and RT-qPCR are listed in Table S4.

### ChIP-seq and ChIP-qPCR.

Formaldehyde was added into RirA-Flag and Δ*rirA*-FecI-Flag cultures in VMM medium containing 37 μΜ FeCl_3_ (OD_590_, 0.6) to a final concentration of 1%. The resultant suspension was kept at room temperature for 12 min with gentle shaking, and the cross-linking process was then stopped by adding glycine to a final concentration of 100 mM and kept for 5 min. The resultant cell pellets were ground into fine powder in liquid nitrogen and resuspended with ChIP buffer (50 mM Tris-HCl, pH 8.1, 150 mM NaCl, 5 mM EDTA, 1% Triton X-100, and 0.1% sodium deoxycholate) supplied with protease inhibitor cocktails (Roche). Cell lysates were sonicated in Bioruptor Pico (Diagenode Inc.) at 4°C for 9 cycles of 20 s on and 30 s off to shear chromatin DNA fragments to an average length of 200 to 300 bp. After centrifugation at 14,000 × *g* for 15 min at 4°C, supernatants were normalized by dilution with ChIP buffer to have a uniform protein content of 2 mg/mL. The supernatant without immunoprecipitation of 30 μL was used for total chromatin input DNA preparation. For each ChIP reaction, 100 μL anti-FLAG M2 magnetic beads (Sigma) prebalanced with TBS buffer (50 mM Tris-HCl, 150 mM NaCl, pH 7.4) was mixed with1 mL diluted supernatant and incubated for 3 h with low-speed rotation at 4°C. The beads were washed three times with TBS buffer before a two-step elution in 500-μL 3×FLAG peptide solution (150 ng/μL in TBS buffer). Eluted protein-DNA complexes were supplemented with 20 μL 5 M NaCl and incubated overnight at 65°C. Samples were further digested with 20 μg RNase A and 50 μg proteinase K for 30 min at 37°C. DNA was purified by using phenol/chloroform/isoamylalcohol (25:24:1) extraction and GenTLE precipitation carrier (TaKaRa)-assisted high-efficiency ethanol precipitation. ChIP DNA samples prepared from RirA-Flag cultures were sent to Novogene-Beijing for library construction (200 to 300 bp) and deep sequencing (paired-end 150-bp reads using NovaSeq6000), with the total chromatin input DNA as control. Two biological replicates were tested.

To determine the recruitment levels of RirA-Flag and FecI-Flag by promoter regions of candidate target genes, ChIP-qPCR was conducted using diluted DNA recovered from input and ChIP samples as the templates. qPCR was performed by using methods same as that used for RT-qPCR as described above. 16S rRNA gene was used as the internal reference. Primers used in ChIP-qPCR are listed in Table S4. Three independent biological replicates were analyzed.

### ChIP-seq data analysis.

Genome-wide mapping of clean ChIP-seq reads was performed by running Bowtie2 to generate BAM files, which were used as input of MACS2 (version 2.1.0) for peak calling (91). The parameters for peak calling are specified as follows: “--call-summits -B -g 6913799 –bw 300 –qvalue 0.05.” Positive peaks (fold enrichment > 2; false-discovery rate [FDR] < 0.01) identified in any of the two replicates were retained and reallocated with the same peak ID when summit distance was less than 200 bp for different replicates. ChIP peaks and associated genes were visualized by using Integrative Genomics Viewer (IGV) software (92). The overrepresented motif of ChIP-seq peaks was determined by analyzing the 200-bp central region (with summit in the peak center) using the web-based MEME-ChIP module (93).

### Phylogeny and ortholog protein analysis.

Neighbor-joining trees were constructed by using MEGA X (94) based on full length of housekeeping RpoB protein to represent as “species tree.” Concatenated protein sequences of AbsABCDEF were used as seed for online TBLASTN analysis against the RefSeq genome database to find out genomes encoding this biosynthetic gene cluster. We selected 24 representative genomes for further phyletic distribution and synteny analysis of genes involved in petrobactin synthesis and transport. The bidirectional best hits (BBH) method was used to identify ortholog candidates, which were further verified by phylogenetic analyses.

### Data availability.

Clean reads data from our ChIP-seq analysis can be accessed via NCBI BioProject (accession no. PRJNA759371).
