# Supplementary material for: Rhizobiales-Specific RirA Represses a Naturally “Synthetic” Foreign Siderophore Gene Cluster To Maintain Sinorhizobium-Legume Mutualism
Source: mBio. 2022 Feb 8;13(1):e02900-21. doi: 10.1128/mbio.02900-21 (PMC8822346; doi:10.1128/mbio.02900-21)
Supplement: FIG S1 [file mbio.02900-21-sf001.pdf]

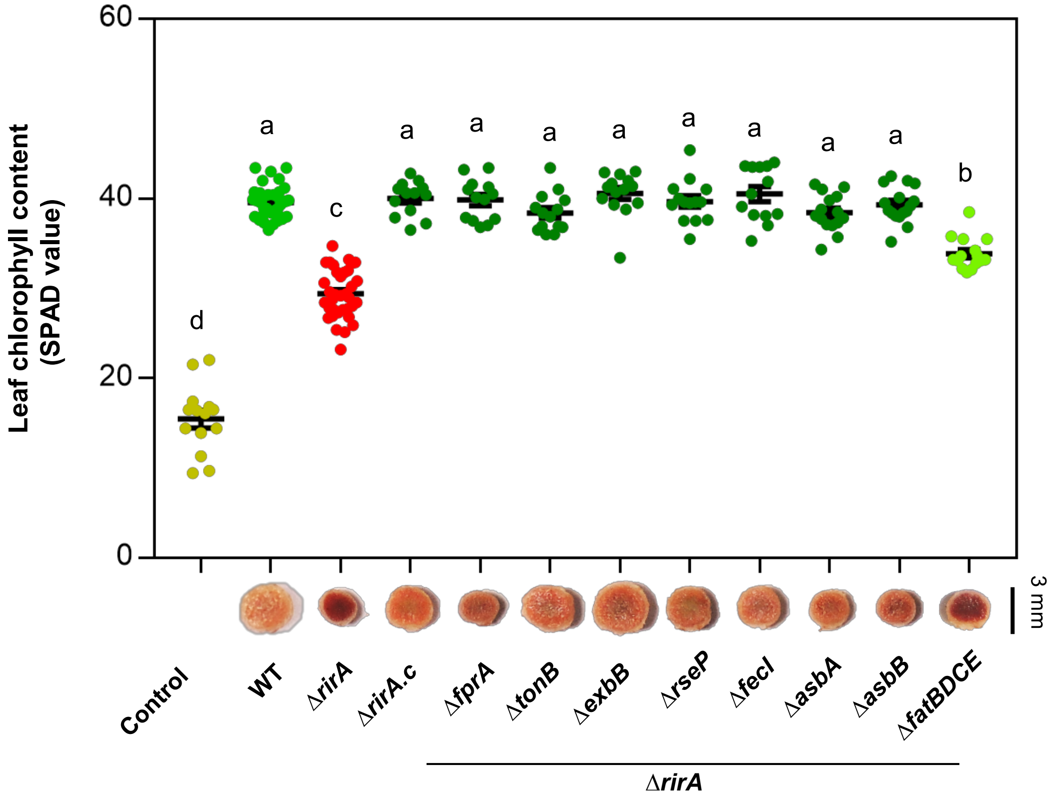

Figure S1. Symbiotic performance of suppressor mutants on wild soybean plants. The combined mutants were obtained by in frame deletion of related genes in the Δ*rirA* background as indicated. The *asbB* and *fatBDCE* were also deleted in the Δ*rirA* background for comparison. Representative pictures of vertical section of nodules are shown. Different letters indicate significant difference between means (mean ± SE; ANOVA followed by Duncan's test, alpha = 0.05) based on more than thirteen scored plants from three independent experiments. Detailed statistics of symbiotic performance for test strains are shown in Table S5.
