# Supplementary material for: Rhizobiales-Specific RirA Represses a Naturally “Synthetic” Foreign Siderophore Gene Cluster To Maintain Sinorhizobium-Legume Mutualism
Source: mBio. 2022 Feb 8;13(1):e02900-21. doi: 10.1128/mbio.02900-21 (PMC8822346; doi:10.1128/mbio.02900-21)
Supplement: FIG S2 [file mbio.02900-21-sf002.pdf]

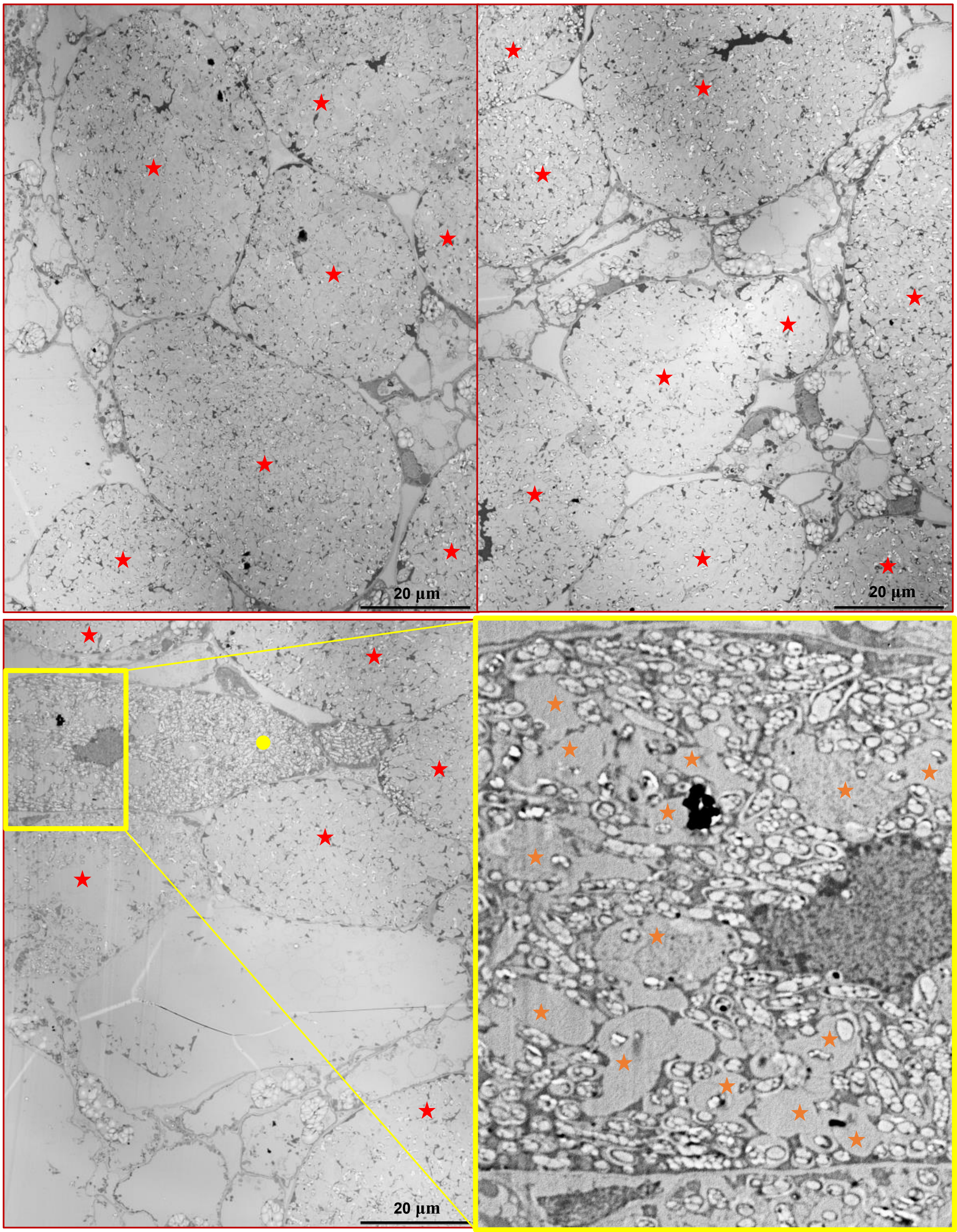

Figure S2. Most nodule cells infected by the Δ*rirA* mutant harboring low bacterial density. Ultrathin sections of nodules (28 dpi) observed with transmission electronic microscopy. Filled yellow circle (only one in three quadrats) and red star indicate two kinds of nodule cells infected by the Δ*rirA* mutant. Orange stars indicate symbiosomes harboring degrading rhizobia (similar to those in red star cells) in nodule cells of normal bacterial density (yellow circle cells).
