# Supplementary material for: Rhizobiales-Specific RirA Represses a Naturally “Synthetic” Foreign Siderophore Gene Cluster To Maintain Sinorhizobium-Legume Mutualism
Source: mBio. 2022 Feb 8;13(1):e02900-21. doi: 10.1128/mbio.02900-21 (PMC8822346; doi:10.1128/mbio.02900-21)
Supplement: FIG S3 [file mbio.02900-21-sf003.pdf]

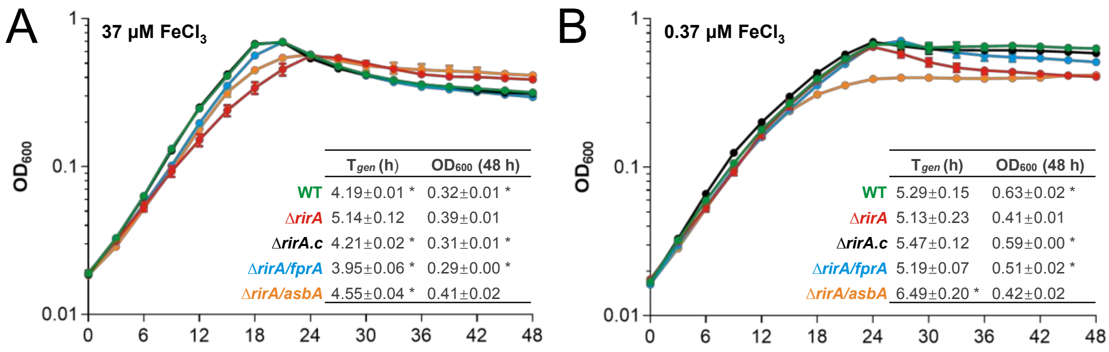

Figure S3. Growth curves and generation time (T*gen*) for test strains. (**A**) the iron replete condition (37 µM $FeCl_3$) and (**B**) the iron deficient condition (0.37 µM $FeCl_3$) were used. Significant difference compared to the Δ*rirA* mutant is indicated (*t* test, *, P-value < 0.05; mean ± SE based on three biological replicates).
