# Supplementary material for: Rhizobiales-Specific RirA Represses a Naturally “Synthetic” Foreign Siderophore Gene Cluster To Maintain Sinorhizobium-Legume Mutualism
Source: mBio. 2022 Feb 8;13(1):e02900-21. doi: 10.1128/mbio.02900-21 (PMC8822346; doi:10.1128/mbio.02900-21)
Supplement: FIG S4 [file mbio.02900-21-sf004.pdf]

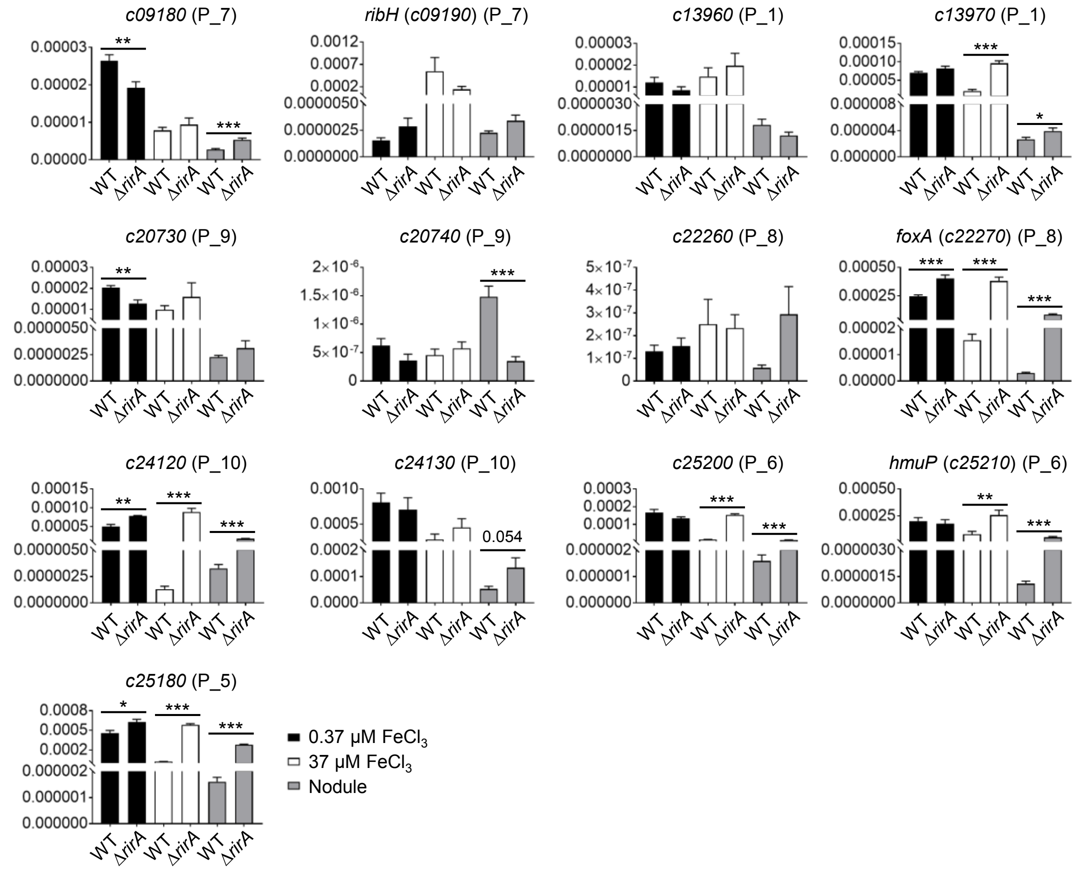

Figure S4. RT-qPCR analyses of RirA targets. Free-living cultures under iron replete (37 μM $FeCl_3$) or deficient (0.37 μM $FeCl_3$) conditions, and rhizobia within soybean nodules (28 dpi). The transcription level relative to 16S rRNA gene is shown. Significant differences are indicated (mean ± SE based on three independent experiments; *t* test, *, P-value < 0.05; **, P-value < 0.01; ***, P-value < 0.001).
