# Supplementary material for: Rhizobiales-Specific RirA Represses a Naturally “Synthetic” Foreign Siderophore Gene Cluster To Maintain Sinorhizobium-Legume Mutualism
Source: mBio. 2022 Feb 8;13(1):e02900-21. doi: 10.1128/mbio.02900-21 (PMC8822346; doi:10.1128/mbio.02900-21)
Supplement: FIG S5 [file mbio.02900-21-sf005.pdf]

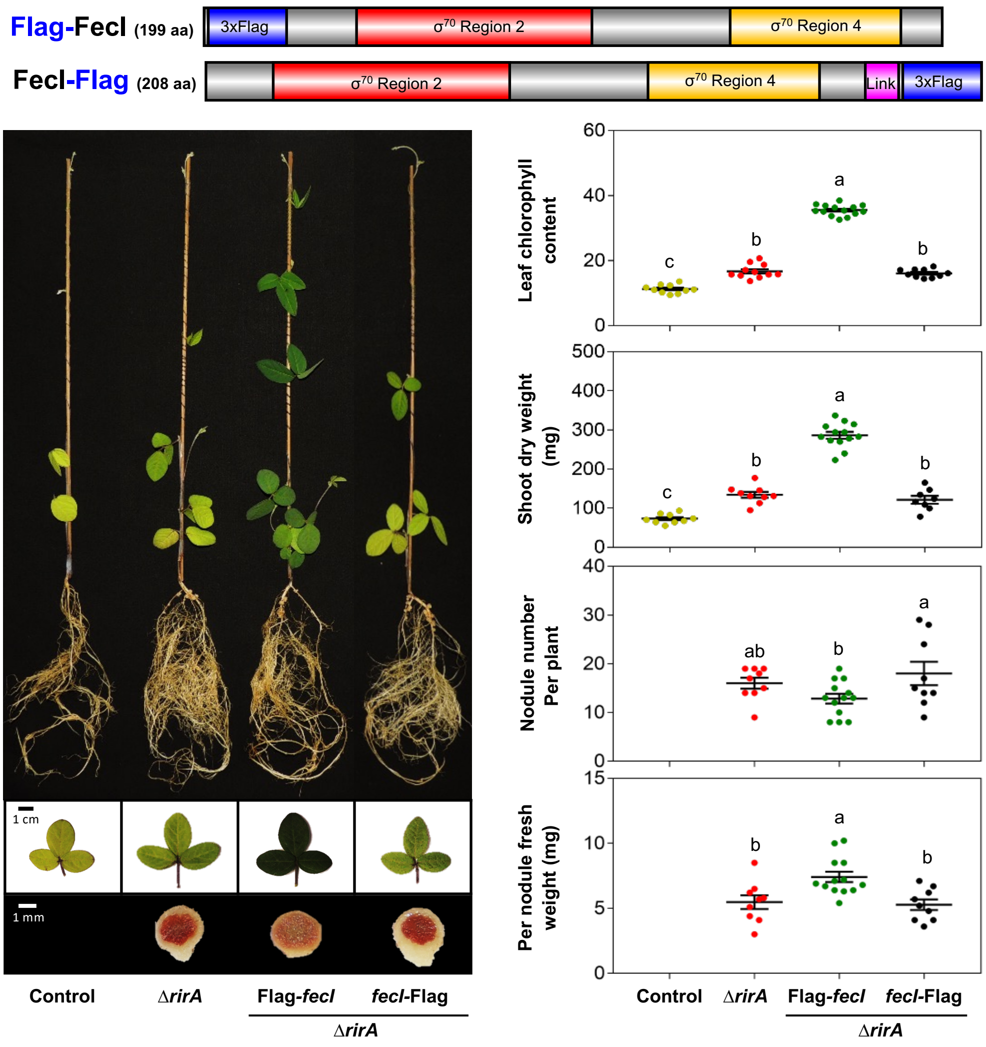

Figure S5. Symbiotic performance of the Δ*rirA* mutant carrying either the non-functional Flag-FecI or functional FecI-Flag. Different letters indicate significant difference between means (mean ± SE; ANOVA followed by Duncan's test, alpha = 0.05).
